# Supplementary material for: Variation in inbreeding depression within and among Caenorhabditis species
Source: bioRxiv. 2025 Jun 7:2025.06.04.657873. Preprint. [Version 1] doi: 10.1101/2025.06.04.657873 (PMC12157486; doi:10.1101/2025.06.04.657873)
Supplement: Supplement 1 — Table S1. SibMatingExperiment.csv Raw data reporting the results of the 1200 sibmating experiments described in the manuscript. For each experiment, the table reports the experiment number (used to blind the experimenters to the strain identity), an identifier for the worm picker who carried out the experiment, and the worm strain and species. The result for each experiment is recorded as the last generation of the experiment in which the strain was observed alive. The status column indicates whether the strain died in that last recorded generation (status = 1) or whether it was still alive (status = 0). The columns Copulation, LaidEmbryos, and Young Adults record for each experiment whether in the final generation the line successfully copulated, laid embryos, and produced offspring that developed to the Young Adult stage. The data in this file underlie Figure 1 and Table 1. Table S2. RegressionResults.csv For each parameter (A, B) and each trait (Reproduction, Copulation, Fertility, Growth), the table reports the estimate, standard error, t-statistic, and p-value for each of the 11 isolates. For inbreeding load B, the table also shows p-values estimated from 10,000 simulations under the null hypothesis of no load. Table S3. RelativeFitnessData.csv For each of 120 individual females tracked through their reproductive lives, the table reports the strain, species, experimental plate, and number of male and female adults that developed from each of eight 8-hour time blocks. For example, columns 1F and 1M record the Female and Male progeny that developed to adulthood from embryos laid during the 1st time block. These data underlie Figure 2. Table S4. ScannerFitnessData.csv For each of 59 experimental plates tracked by flatbed scanner for population growth, the table records the genotype (treatment), the phenotype (sd_hours_to_starve, hours to resource exhaustion, measured as the peak of the standard deviation of pixel intensity), the replicate plate for the treatment ( [file media-1.zip › ReviewSupplement/FileS1.Model.pdf]

Variation in inbreeding depression within and among *Caenorhabditis* species

File S1:
Comparison of reproduction probability to Morton *et al.*'s survival probability

Morton *et al.* model the probability of survival (S) given a single recessive locus as $S = 1 - Fqs - (1\text{-}F)q^2s - (1\text{-}F)2pqsh,$ where s is the probability that an individual homozygous for the recessive allele fails to survive, q and p are the mutant allele frequency and its complement, h is the dominance coefficient, and F is the inbreeding coefficient. We substitute mating success for survival, and model the probability that two worms successfully mate, which requires that both worms succeed. Thus the probability of successful reproduction (R) is the product of the two worms' individual reproduction probabilities (i.e., $R = S^2$), yielding $R = 1 - 2Fqs + F^2q^2s^2 - 2(1\text{-}F)q^2s + 2F(1\text{-}F)q^3s^2 - 4(1\text{-}F)pqsh + 4F(1\text{-}F)pq^2s^2h + (1\text{-}F)^2q^4s^2 + 4(1\text{-}F)^2pq^3s^2h + 4(1\text{-}F)^2p^2q^2s^2h^2$.

In this case, unlike that of Morton *et al.,* the probability is quadratic rather than linear as a function of F. However, as shown below, all of the F^2 terms include q^2s^2 and are negligible.

Morton *et al.* extended their model to include the effects of multiple loci and environmental causes of individual failure (x). Assuming that these causes act independently, $S = \prod(1\text{-}x)(1 - Fqs - (1\text{-}F)q^2s - (1\text{-}F)2pqsh)$, where the product is over all loci and environmental causes. Using the approximation $1–t \sim e^{-t}$ for small $t,$ and assuming that environmental effects x act independently on the two animals in each cross,
$R = \exp(-2\sum x + \sum x^2 - 2\sum q^2s - 4\sum pqsh + \sum q^4s^2 + 4\sum pq^3s^2h + 4\sum p^2q^2s^2h^2 - 2F\sum qs + 2F\sum q^2s + 2F\sum q^3s^2 + 4F\sum pqsh + 4F\sum pq^2s^2h - 2F\sum q^4s^2 - 8F\sum pq^3s^2h - 8F\sum p^2q^2s^2h^2 + F^2\sum q^2s^2 - 2F^2\sum q^3s^2 - 4F^2\sum pq^2s^2h + F^2\sum q^4s^2 + 4F^2\sum pq^3s^2h + 4F^2\sum p^2q^2s^2h^2)$

Thus,
$-\log R = A_R+B_RF+C_RF^2$
where $A_R = 2\sum x - \sum x^2 + \sum qs(2q + 4ph - q^2s - 4pq^2sh - 4p^2qsh^2)$
$B_R = 2\sum qs(1 - q - q^2s - 2ph - 2pqsh + q^3s + 4pq^2sh + 4p^2qsh^2)$
and $C_R = \sum q^2s^2(-1 + 2q + 4ph - q^2 - 4pqh - 4p^2h^2)$.

B_R and C_R are both zero when $h = 0.5$. The quadratic coefficient C_R is much less than 1% of B_R for any plausible values of $s, h,$ and q.

The coefficients A_R and B_R here are approximately twice the magnitude of those in the single-individual case of Morton et al.
$B_{Survival} = \sum qs(1 - q - 2ph)$
$B_R = 2B_{Survival} + 2\sum q^2s^2(-q - 2ph + q^2 + 4pqh + 4ph^2)$

Similarly,
$A_{Survival} = \sum x + \sum qs(q+ 2ph)$
$A_R = 2A_{Survival} - \sum x^2 - \sum q^2s^2(q + 4pqh + 4p^2h^2)$
